# Supplementary material for: Pedagogical strategies for teaching nursing theories in undergraduate students: A scoping review protocol
Source: PLoS One. 2024 Oct 16;19(10):e0308783. doi: 10.1371/journal.pone.0308783 (PMC11482667; doi:10.1371/journal.pone.0308783)
Supplement: Attachment — Submitted filename: Response to Reviewers.docx [file pone.0308783.s002.docx]

Sébastien Colson and Aurélie Demagny-Warmoes

Aix-Marseille Univ, APHM, CEReSS, Faculty of Medical and Paramedical Sciences, Nursing School, Marseille, France

E-mail:

[sebastien.colson@ap-hm.fr](mailto:sebastien.colson@ap-hm.fr)

[aurelie.warmoes@etu.univ-amu.fr](mailto:aurelie.warmoes@etu.univ-amu.fr)

Marseille, July 23, 2024

Dear Dr. Chenette,

We are extremely grateful for your interest in our paper and for the constructive review of our manuscript. Please find enclosed the revised version of our article entitled: *“Pedagogical strategies for teaching nursing theories in undergraduate students: a scoping review protocol”.*

We are pleased to inform you that we have received funding approval for this article from Assistance Publique – Hôpitaux de Marseille (AP-HM), which has entered into a partnership with PLOS ONE. Additionally, please note that the corresponding author for this manuscript is now Professor Sébastien Colson, the last author of the article.

In line with the reviewers’ and the editor’s comments, we have modified the manuscript in order to improve the quality of presentation and content in general.

Note: Please see the attached 'Point-by-point response letter' in which a colour code has been added to distinguish between our responses and the comments of the reviewers.

**-Point-by-point response letter-**

# Response to Editor

We thank Editor for comments and constructive suggestions. We have carefully revised the manuscript according to each comment and suggestion.

Colour code:

- In red: Our response.
- In green: the line number in the current reviewed version of the manuscript.

**Point 1: PLOS ONE's style requirements**

Line: 1,2,3,16,57,63,78,91,98,124,134,165,176,191,201,206,227,239.

All titles have been formatted in Times New Roman size 18, and the subtitles in Times New Roman size 16. Line: 102,108,116.

Line: 58,64,79,92,99,103,109,117,125,135,166,192,202,207,228. An indent has been added at the beginning of each paragraph.

Line: 153, 162. The titles of tables 1 and 2 now comply with the editorial guidelines.

Line: 4 to 13. The abstract has been written in accordance with the editor's recommendations.

**Point 2: Data Availability Statement**

This protocol has been registered on the Open Science Framework website. It is currently under restricted access until the article is published. Here is the registration link:

<http://osf.io/gj35n>

### Point 3: Billing option

### The manuscript has been revised to change the corresponding author. The new corresponding author is Professor Sébastien Colson at the following address: [sebastien.colson@ap-hm.fr](mailto:sebastien.colson@ap-hm.fr). Professor Colson, the last author of this article, is affiliated with APHM, which subsidizes the publication fees. Affiliations 2 and 3 have been modified as well.

Point 4: Information files

No supplementary information file

**Point 5: Reference list**

As requested by the reviewer, references 12 and 15 have been modified.

Reference 12 : Peters M, Gogfrey C, Mclnerney P, Munn Z, Tricco A, Khalil H. Chapter 11: Scoping reviews. In: Aromataris E, Munn Z (Editors) JBI Manuel for Evidence Synthesis, JBI [Internet]. 2020. Available from: https://synthesismanual.jbi.global. <https://doi.org/10.46658/JBIMES-20-12>

Line: 262 to 265. Replaced by:Peters MDJ, Gogfrey C, Mclnerney P, Munn Z, Tricco AC, Khalil H. Scoping Reviews. In: Aromataris E, Lockwood C, Porritt K, Pilla B, Jordan Z (Editors) JBI Manuel for Evidence Synthesis, JBI; 2024. Available from: https://synthesismanual.jbi.global. <https://doi.org/10.46658/JBIMES-24-09>

Reference 15: Peters MDJ, Marnie C, Tricco AC, Pollock D, Munn Z, Alexander L, et al. Updated methodological guidance for the conduct of scoping reviews. JBI Evid Implement. 2021 Mar;19(1):3.

Line: 272 to 275. Replaced by Aromataris E, Lockwood C, Porritt K, Pilla B, Jordan Z.JBI Manual for Evidence Synthesis.JBI.2024. <https://doi.org/10.46658/JBIMES-24-01>

Line: 88. Since we have included more recent references, we had to change chapter 11 to chapter 10.

# Response to Reviewer

We thank Reviewer for comments and constructive suggestions. We have carefully revised the manuscript according to each comment and suggestion.

Colour code:

- In red: Our response.
- In green: the line number in the current reviewed version of the manuscript.

**Point 1:**

Please consider to review the keyword: "scoping review protocol", because it is not MESH term.

Line: 14. Indeed, "scoping review protocol" does not exist in MeSH. We propose to remove it. Since the MeSH term "protocol" primarily represents clinical research protocols, we prefer to remove this term and replace it with the MeSH term "models, nursing" to meet the recommended number of keywords.

**Point 2:**

Please consider to check:
- Aromataris E, Lockwood C, Porritt K, Pilla B, Jordan Z, editors. JBI Manual for Evidence Synthesis. JBI; 2024. Available from: <https://synthesismanual.jbi.global>. <https://doi.org/10.46658/JBIMES-24-01>
- Peters MDJ, Godfrey C, McInerney P, Munn Z, Tricco AC, Khalil, H. Scoping Reviews (2020). Aromataris E, Lockwood C, Porritt K, Pilla B, Jordan Z, editors. JBI Manual for Evidence Synthesis. JBI; 2024. Available from: <https://synthesismanual.jbi.global>. <https://doi.org/10.46658/JBIMES-24-09>

As requested by the reviewer, references 12 and 15 have been modified.

Line: 262 to 265. Replaced reference 12 by: Peters MDJ, Gogfrey C, Mclnerney P, Munn Z, Tricco AC, Khalil H. Scoping Reviews. In: Aromataris E, Lockwood C, Porritt K, Pilla B, Jordan Z (Editors) JBI Manuel for Evidence Synthesis, JBI; 2024. Available from: https://synthesismanual.jbi.global. <https://doi.org/10.46658/JBIMES-24-09>

Line: 272 to 275. Replaced reference 15 by: Aromataris E, Lockwood C, Porritt K, Pilla B, Jordan Z.JBI Manual for Evidence Synthesis.JBI.2024. <https://doi.org/10.46658/JBIMES-24-01>
